# Metabolic Disruption by Naturally Occurring Mycotoxins in Circulation: A Focus on Vascular and Bone Homeostasis Dysfunction

**DOI:** 10.3389/fnut.2022.915681

**Published:** 2022-06-24

**Authors:** Amir Mohammad Malvandi, Sara Shahba, Jalil Mehrzad, Giovanni Lombardi

**Affiliations:** ^1^Laboratory of Experimental Biochemistry and Molecular Biology, IRCCS Istituto Ortopedico Galeazzi, Milan, Italy; ^2^Department of Microbiology and Immunology, Faculty of Veterinary Medicine, University of Tehran, Tehran, Iran; ^3^Department of Athletics, Strength and Conditioning, Poznań University of Physical Education, Poznań, Poland

**Keywords:** mycotoxins, low dose toxicity, oxidative stress, metabolic and immune alterations, bone homeostasis, cardiovascular diseases

## Abstract

Naturally occurring food/feed contaminants have become a significant global issue due to animal and human health implications. Despite risk assessments and legislation setpoints on the mycotoxins' levels, exposure to lower amounts occurs, and it might affect cell homeostasis. However, the inflammatory consequences of this possible everyday exposure to toxins on the vascular microenvironment and arterial dysfunction are unexplored in detail. Circulation is the most accessible path for food-borne toxins, and the consequent metabolic and immune shifts affect systemic health, both on vascular apparatus and bone homeostasis. Their oxidative nature makes mycotoxins a plausible underlying source of low-level toxicity in the bone marrow microenvironment and arterial dysfunction. Mycotoxins could also influence the function of cardiomyocytes with possible injury to the heart. Co-occurrence of mycotoxins can modulate the metabolic pathways favoring osteoblast dysfunction and bone health losses. This review provides a novel insight into understanding the complex events of coexposure to mixed (low levels) mycotoxicosis and subsequent metabolic/immune disruptions contributing to chronic alterations in circulation.

## Introduction

Mycotoxins are small, highly stable toxic molecules contaminating feed and food chains (1). They are classified into the main categories of carcinogenic substances, including aflatoxins (AFs), ochratoxins, fumonisins, trichothecenes, and zearalenone (ZON) (2). Each mycotoxin is considered a secondary metabolite naturally produced by various fungi genera, *Aspergillus, Penicillium, Fusarium, Alternaria*, and *Claviceps*. The lack of quality control, climate change, intensive farm production, and storage technologies might favor a more frequent mycotoxin occurrence even in Europe/western countries (3). Although regulatory limits on significant levels are established in many countries, they remain a concerning issue due to the continuous exposure, although at very low concentrations.

Both acute and chronic exposure to mycotoxins can lead to teratogenic, mutagenic, carcinogenic, nephrotoxic, hepatotoxic, and immunotoxic effects on different organs (4). We recently reviewed the impact of naturally occurring mycotoxins dysregulating the immune system in the brain (5). However, knowledge regarding the consequences of exposure to low-level mycotoxins on circulation is modest. Circulating cells, bone marrow niche, and vascular tissue are easy targets of mycotoxins' toxicity (Table 1). There is no safe level; synergistic interaction of nanomolar concentrations of common mycotoxins could cause serious problems compared to higher concentrations (39).

**Table 1:** The metabolic and immune shifts induced by mycotoxins (focusing on low concentrations).

**Mycotoxin**	**Effective dose, target**	**Effect**	**Major conclusion**	**References**
AFB_1_	≤ 25 μg/kg, male F344 rat	↑ Percentages of CD3^+^ and CD8^+^ T cells ↓ IL-4	5-week exposure modulates the cell-mediated immune responses	(6)
	30 μg/kg, murine	↑ Protein and DNA synthesis in splenic lymphocytes, selectively affects helper T cells	Alteration in host immunity by repeated treatment for 4 weeks	(7)
	60, 300, and 600 μg, rat	↓ DTH response (300 and 600) ↓ Proliferative response to PHA (60, 300, and 600) Inhibition of lymphocyte proliferation	Continuous low levels suppress cell-mediated immunity and high susceptibility to infections and tumorigenesis	(8)
	20 ng/mL, human microglia cell line	Overexpression of TLRs, MyD88, NF-κB, IKβ kinase, CXCR4, CCR4, and CCR8 Induction of intracellular ATP depletion Caspase-3/7 activation IFN-γ and GM-CSF secretion ↑ Apoptosis	Alteration in key factors related to inflammation in vital immune-keeper cells	(9)
	10 ng/mL, porcine monocyte-derived DCs	Dysregulation of the antigen-presenting capacity of DCs	Immunosuppressive effects on APCs and thus naïve T cells	(10)
	AFB_1_ (16.3–134 μg/kg feed) AFB_2_ (3.15–23.6 μg/kg feed), broiler	Disruption in cells cycle progression and apoptosis Histopathological lesions in thymus and bursa fabricius	Disturbed T and B lymphocytes maturation	(11)
	0.6 mg/kg, broilers chicken	Diffused intestinal epithelial cells Disappearance of microvilli, mitochondrial vacuolation, and mitochondrial cristae ↓ TLR-2/4/7 expression	↓ Absorptive capacity of the small intestine (ultrastructural changes) Impairment of innate immunity of the small intestine	(12)
	0.6 mg/kg, broilers chicken	↓ Percentage of T-cell subsets ↓ mRNA expression levels of IL-2, IL-4, IL-6, IL-10, IL-17, IFN-γ, and TNF-α in the small intestinal mucosa	Intestinal mucosal immunity in the duodenum, jejunum and ileum	(13)
	0.1 pg/mL, human monocyte	↓ Phagocytosis and microbicidal activity of monocytes	Induction of depressed monocytes and high susceptibility to infections	(14)
	5.10e-11 M or 0.05 μg/L, human NK cell	↓ Cytotoxic and proliferative activities evaluated by a 51Cr release NK assay	Immunosuppressive effects on NK cells	(15)
	0.11 to 0.21 mg/kg, duckling	↓ Serum glucose, creatinine, albumin, total protein, globulin, Ca, P, and CPK ↑ Serum urea N, Cl, ALP, and AST levels ↓ L_100_ and L_50_ values (lysis titer) for rabbit, human, and horse erythrocytes ↓ Rabbit HA1 value (strong hemagglutination) ↓ PPARα expression level	Liver damage Alteration of serum proteins and enzyme activity Impairment of innate immunity by reducing natural antibody and complement activities	(16)
	140 and 280 μg/L, weanling piglet	↓ Total number of white blood cells ↑ Serum gamma-globulin ↓ IL-1β and TNF-α ↑ IL-10 mRNA expression	Alteration of many aspects of humoral and cellular immunity	(17)
	5–80 nM, BEAS-2B cell line human	↑ C-PARP, C-caspase-3, and Bax expression ↓ Caspase-3, Bcl-2, and p-Bad expression ↑ DNA adduct and damage Activation of ATM, ATR, Chk2, p53, BRCA1, and H2AX proteins	Cytotoxic and apoptotic effects on immortalized human bronchial epithelial cells mediated by cytochrome P450 2A13	(18)
AFM_1_	3.2 and 33 nM, Caco-2/TC7 cell	↓ Value of trans-epithelial electrical resistance	Acceleration of AFs transport	(19)
	25 or 50 μg/kg, murine (Predicted no observable effect level (NOAEL) is estimated to be 2.5 μg/kg)	↓ Spleen and thymus mass ↓ Hemagglutination titer ↓ Spleen cellularity ↓ Proliferation response to LPS and PHA ↓ CH50 ↓ DTH response ↓ Spleen cell subtypes ↓ Serum IgG level and ↓ IFN-ɤ ↑ IL-10	Suppression of innate and acquired immunity	(20)
OTA	3 μM, macrophagic cell line, J774A.1	↑ COX-2 and iNOS expression ↓ COX-2 and iNOS expression, co-stimulated with LPS ↑ PGE_2_ release and ↓ PGE_2_ release (LPS) ↑ NO production and ↓ NO production (LPS) ↓ cytosolic IκBα level (time-dependent) ↑ p65 NF-κB expression (time-dependent)	Interfering with inflammatory responses against LPS-containing pathogens	(21)
	1 μg/mL, blood lymphocytes of broiler chickens	↑ MDA levels ↑ Acetylcholinesterase enzymatic activity	↑ Cellular oxidative stress levels Disturbing lymphocytes activation and differentiation	(22)
FB_1_	8 mg/kg, weanling piglet	In males, ↓ mycoplasma-specific antibody levels and T helper2 cytokines (IL-10) mRNA expression level after vaccination	Sex-related immunosuppressive effects	(23)
T-2 Satratoxin	200 μg/kg, porcine ileal Peyer's patches	↓ IL-10 production ↓ IL-4 and IFN-γ (not significant) ↑ Percentage of CD8^+^ T lymphocytes (days 14 and 42) ↓ Percentage of CD8^+^ T lymphocytes (day 28) ↓ Percentage of CD21^+^ B cells ↓ Percentages of CD4^+^ and CD8^+^ T lymphocytes (days 14 and 28)	Chronic exposure to low doses affects lymphocytes-mediated humoral immune responses	(24)
	≤ 10 ng/mL RAW 264.7 murine macrophage and U937 human leukemic cells	Induction of apoptosis ERK1/2, p38MAPK, and SAPK/ JNK activation in myeloid models	Alteration in leukocytes viability and function	(25)
DON	1 mg/L and 0.2 mg/L, mice	↓ Specific IgM titer and lower DTH reaction Inhibition of a cell-mediated immune response	↓ Resistance against Salmonella infections through toxic effects on cellular and humoral immunity	(26)
	Up to 500 ng/mL, human B (RPMI1788) and T (Jurkat E6.1) lymphocyte cell lines	↓ Cells viability (at 250 and 500 ng/mL) Alteration of phosphorylation state in proteins: ↑C1-THF synthase, ↑eEF2, ↓GRB2, ↑eIF3i, ↑NDKA, and ↓HSC70, (involved in immune functions with metabolism regulation, protein biosynthesis, co-chaperoning, and signaling transduction)	Phosphoproteomic changes in human T and B lymphocytes	(27)
	2.2–2.5 mg/kg, pig	↑ Total IgA plasmatic levels (47% increase) ↑Specific IgA production (160% increase) Biphasic effects, increase and decrease in lymphocyte proliferation ↓IFN-ɤ and TGF-β mRNA expression in mesenteric lymph nodes	Disruption in vaccine immune response	(28)
	1.2–2 mg/kg, pig	↓ IL-1β, IL-8, and TNF-α in blood and ileum	Chronic exposure induces down-regulation of immune-related factors	(29)
	1 and 2 mg/kg, BALB/c mice	lymphoid inhibition ↓ Relative number of mononuclear cells ↓ Percentage of B cells (CD19^+^) in blood Inhibition of CD19^+^ progenitor or newly B cells in bone marrow	Lower humoral and innate immunity (with a reduction in B cells and monocytes), especially in infectious conditions	(30)
		↓ Monocytes in blood and spleen in BALB/c female mice Delay in monocyte or macrophage maturation		
	≤ 10 μmol/L, human epithelial cell line (HT-29-D4)	↓ Human intestinal epithelial cells proliferation ↓ D-glucose/D-galactose sodium-dependent transporter SGLT1, sugar transporters: GLUT5, and L-serine transporter ↑ water absorption in the intestinal lumen	Induction of apoptosis in intestinal epithelial cells	(31)
	1, 2.5, and 25 mg/kg, mice	Systemic increase in plasma IL-1β concentration Modulation of peripheral organ inflammation biomarkers in brain, liver, duodenum, and adipose tissues ↑ TNF-α, IL-1β, and mPGES-1 mRNA expression level ↓ mPGES-1 mRNA expression level	Sub-chronic exposure to low doses makes a central and peripheral low grade inflammation	(32)
	200 ng/mL, human lymphocyte	50% inhibition of lymphocytes proliferation ↑ IL-2, IFN-γ, and ↓ IL-6 cytokines production by lymphocytes	Considerable effects on human lymphocyte cytokine production	(33)
	1 mg/kg, mice	First, rapidly induction of three MAPK families; JNK1/2, ERK1/2, and p38 phosphorylation in murine spleen Second, AP-1, C/EBP activation Third, prolonged activation of AP-1, CREB, and NF-κB Down-regulation and or up-regulation of TNF-α, IL-1β, and IL-6	Time-dependent dysfunctional effects on immune pathways; kinase signaling pathways and transcription factors	(34)
	≤ 5 μg/mL, Caco-2 cell	↑ IκB phosphorylation and NF-κB activation ↑ IL-1β-induced IL-8 secretion by human intestinal epithelial cells depending on PKR, NF-κB, and MAPK p38 activation	Exacerbating intestinal inflammation	(35, 36)
ZON	8 μg/kg, porcine ileal Peyer's patches	↓ IL-2 and IFN-γ secretion ↑ IL-4 and IL-10 secretion Shifting Th1/Th2 balance toward humoral immune response ↑ B1 cell populations ↓ NK cells proliferation and IFN-γ secretion	Changing lymphocyte phenotypes and impairment of T cell-dependent humoral immune responses	(37)
	8 μg/kg, porcine ileal Peyer's patches	↑ IL-4 and IL-10 concentrations Shifting polarization toward Th2 cells and stimulation of B cells ↑ IL-2 and IFN-γ cytokine levels (not significant)	Changes in Th1/Th2 immune responses and susceptibility to autoimmune (development of allergies) and infectious diseases	(38)

*AFB_1_, aflatoxin B_1_; CD3, cluster of differentiation 3; IL-4, interleukin 4; DTH, delayed type of hypersensitivity; PHA, phytohemagglutinin; TLR, toll-like receptor; MyD88, myeloid differentiation primary response 88; NF-κB, Nuclear Factor-Kappa B; CXCR4, C-X-C chemokine receptor type 4; CCR4, C-C chemokine receptor type 4; ATP, adenosine triphosphate; IFN-γ, interferon gamma; GM-CSF, granulocyte-macrophage colony-stimulating factor; DCs, dendritic cells; APCs, antigen-presenting cells; TNF-α, tumor necrosis factor alpha; NK cell, natural killer cell; Ca, calcium; P, phosphor; CPK, creatine phosphokinase; ALP, alkaline phosphatase; AST, aspartate transaminase; HA1, strong hemagglutination; PPARα, peroxisome proliferator-activated receptor alpha; C-PARP, cleaved poly-ADP-ribose polymerases; Bax, Bcl-2-associated X; Bcl-2, B-cell lymphoma 2; ATM, ataxia-telangiectasia-mutated; ATR, ataxia telangiectasia and Rad3-related; Chk2, checkpoint kinase 2; BRCA1, breast cancer type 1; H2AX, H2A histone family member X; LPS, lipopolysaccharides; CH50, hemolytic complement; IgG, immunoglobulin G; OTA, ochratoxin A; COX-2, cyclooxygenase-2; iNOS, inducible nitric oxide synthase; PGE_2_, prostaglandin E_2_; NO, nitric oxide; IκBα, nuclear factor of kappa light polypeptide gene enhancer in B-cells inhibitor, alpha; MDA, malondialdehyde; FB_1_, fumonisin B_1_; ERK, extracellular signal-regulated kinase; MAPK, mitogen-activated protein kinase; SAPK, stress-activated kinase; JNK, c-Jun N-terminal kinase; DON, deoxynivalenol; C1-THF, C1-tetrahydrofolate; eEF2, eukaryotic elongation factor 2; GRB2, growth factor receptor-bound protein 2; eIF3i, eukaryotic translation initiation factor 3 subunit I; NDKA, nucleoside diphosphate kinase A; HSC70, heat shock cognate 71 kDa protein; TGF-β, transforming growth factor beta; SGLT1, sodium/glucose cotransporter 1; mPGES-1, microsomal prostaglandin E synthase-1; AP-1, activator protein 1; C/EBP, CAAT (Controlled Amino Acid Therapy)/enhancer binding protein; CREB, cAMP response element-binding protein; PKR, protein kinase R; ZON, zearalenone*.

Generation of reactive oxygen species (ROS) occurs with normal cellular metabolism; however, it can also result from exposure to naturally occurring mycotoxins (40). ROS leads to alteration of cell components, including DNA breaks, protein cross-links, lipid peroxidation, and consequent impairment of cell function and hemostasis (41, 42). Although cells are endowed with endogenous antioxidant agents [e.g., superoxide dismutase (SOD), catalase, glutathione peroxidase (GPx)], the deleterious effects of mycotoxin-mediated ROS could overcome these defense mechanisms. Oxidative stress is an imbalance between oxidants-free radicals and other reactive species and antioxidants (41). The accumulation of oxidized molecules also accelerates reactive oxygen intermediates' generation; this would be the overall manifestations of disturbance to redox potential and the main cellular processes.

Accordingly, oxidative damage is assumed to be the underlying mechanism of various molecular injuries, such as immune and metabolic disruptions contributing to cancer's chronic inflammatory phenotype profile, neurodegeneration, and vascular diseases. Indeed, this damage would explain how mycotoxins toxicity at low levels may determine harmful effects on the vasculature. Considering the contribution of low levels of mycotoxins to oxidative stress, a series of activated immune cells, endothelial dysfunctions, oxidized lipoproteins, and recruiting additional reinforcements as crucial players in crosstalk between oxidative imbalance and vascular inflammation can be pretty predictable. Paralleling the evidence, the dysregulated lipid metabolism and the lowered availability of lipid-soluble vitamins with exposure to mycotoxins direct the pressure on osteocytes to release the calcium into the circulation, thus moving toward osteoporosis in a long-term perspective (43). Besides, the high mitochondrial activity in the cells (i.e., cardiomyocytes, hematopoietic cells) makes them more fragile to redox imbalance (44, 45). Therefore, cardio-specific disruptions can be another target of mycotoxins-mediated oxidative damage. This review provides a novel perspective on the oxidative aspects of relatively low-dose mycotoxins in the framework of systemic risk and vascular function.

## Oxidative Stress, Mycotoxin Path to Cell's and System's Dysfunction

Obstruction of the arteries (supplying blood to different tissues), defined as arterial dysfunction, is mainly the buildup of oxidative reactions and calcium and inflammatory deposits restoring each other (46). Tumor necrosis factor (TNF) inhibitors, the most common biologics used in treating rheumatological diseases, would exert favorable effects on the pathophysiology of the blood vessels (47). However, sustained and chronic inflammation is quickly becoming a controversial issue. Recent research has revealed a rich field for further investigation: a dual challenge of integrated oxidative and immune disruptions in circulating cells. There is a fundamental question: how repeated episodes of oxidative stress can induce a persistent inflammatory phenotype?

A significant consequence of the enhanced ROS formation and prolonged oxidative stress exposure is endothelial dysfunction via scavenging of NO by superoxide. The vascular microenvironment and the associated endothelial dysfunction look to predict the prognosis of the lesions. Endothelial cells (ECs) are a source of $O_{2}^{-}$ production contributing to chronic inflammatory states (48). EC's phenotype becomes activated through interaction with leukocytes and platelets, the expression of several proinflammatory cytokines [TNFα and interleukin-1 (IL-1)], and adhesion molecules. NF-κB is the pathway regulating endothelium-leukocyte interactions by adhesion molecules, such as intercellular adhesion molecule-1 (ICAM-1), vascular cell adhesion molecule-1 (VCAM-1), and E-selectin (49). Activation of the NF-κB pathway leads to the inflammatory EC responses by chemokines release, such as macrophage chemoattractant protein-1 (MCP-1) and IL-8 (50). That is, the activated ECs modulate inflammatory cells to overexpress scavenger receptors (CD36) and TLRs, essential for vascular oxidative stress (51).

In addition, ECs are a primary target of oxidative signals in circulation, importantly oxidizing lipoproteins. In the vessel wall, impairment of lipid metabolism could cause pathologic lipids accumulation (52). Malondialdehyde (MDA) represents an important factor in atherosclerosis because it principally exists in low-density lipoprotein cholesterol (LDL-C). Hence, excessive oxidation can act on lipids determining lipid peroxidation products. Indeed, MDA levels could precisely indicate the severity of lipid peroxidation injury, one of the initiating links of endothelial dysfunction in atherosclerosis (53). The oxidative modification of low-density lipoprotein (LDL) is an important inducer for atherogenic and proinflammatory pathways. In the atherosclerotic lesions, the primary source of chronic inflammatory responses is represented by lipid-laden foam cells caused by excessive lipid accumulation, ROS generation, intramural retention of oxidized LDLs (oxLDLs), and minimally oxLDLs, with many of these responses mediated by toll-like receptor-4 (TLR-4, by recognition and assembly of multi-molecular complexes, reviewed elsewhere) (54).

**Figure 1:**
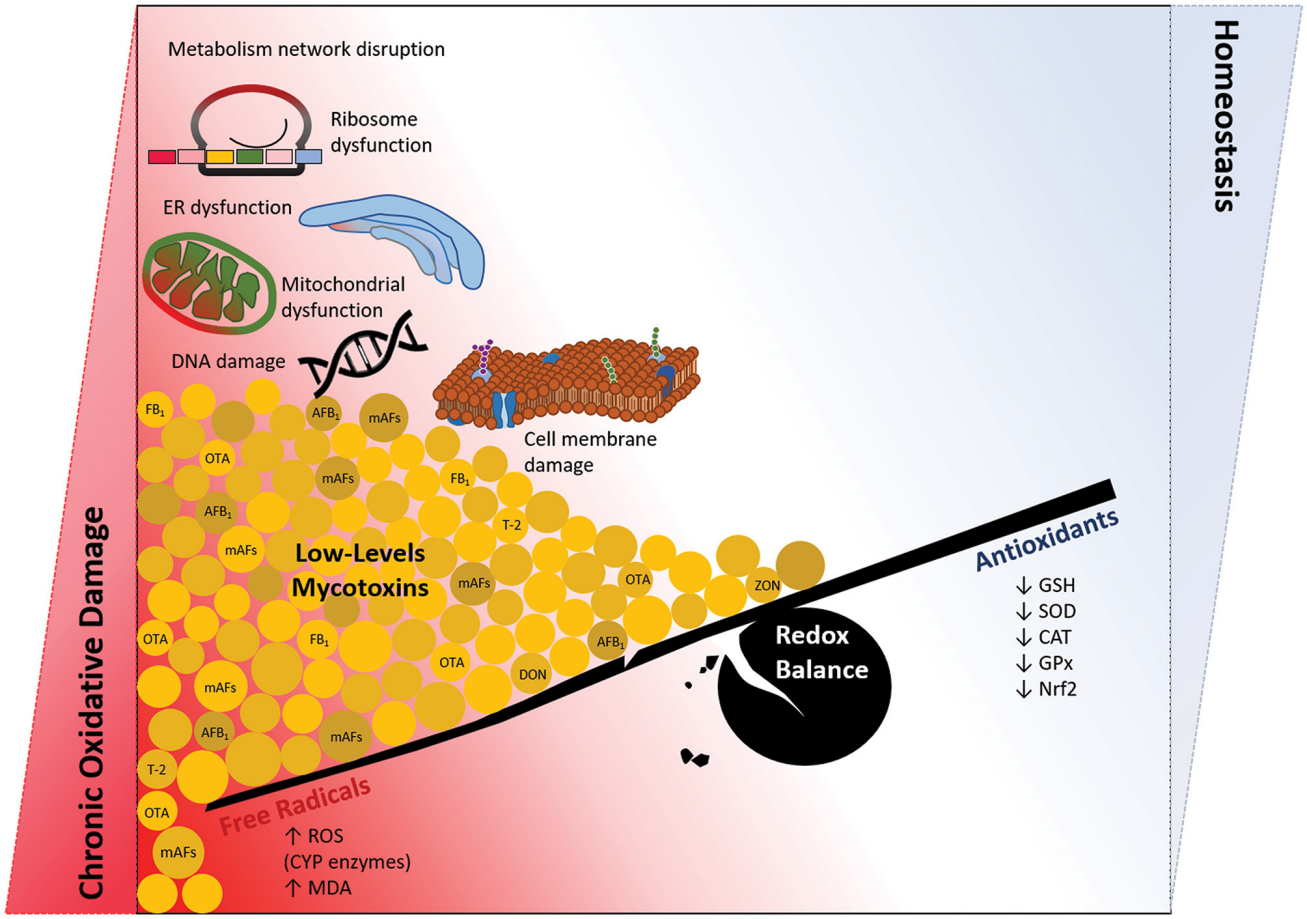
Long-term exposure to foods and feed contaminated by low doses of mycotoxins; the chronic oxidative damage vs. hemostasis. Coexposure to the mixture of mycotoxins, especially at nanomolar doses below the allowable levels (40), induces a chronic condition of oxidative disturbance in the cells. There would not be the context of redox balance regarding a continuous production of free radicals and loss of antioxidants defense system. Then, significant damage to macromolecules (DNA, protein, and lipid), organelles (mitochondria, ER, and ribosome), and metabolism network in the cell may orchestrate several pathophysiological conditions, including cardiovascular damages.

**Figure 2:**
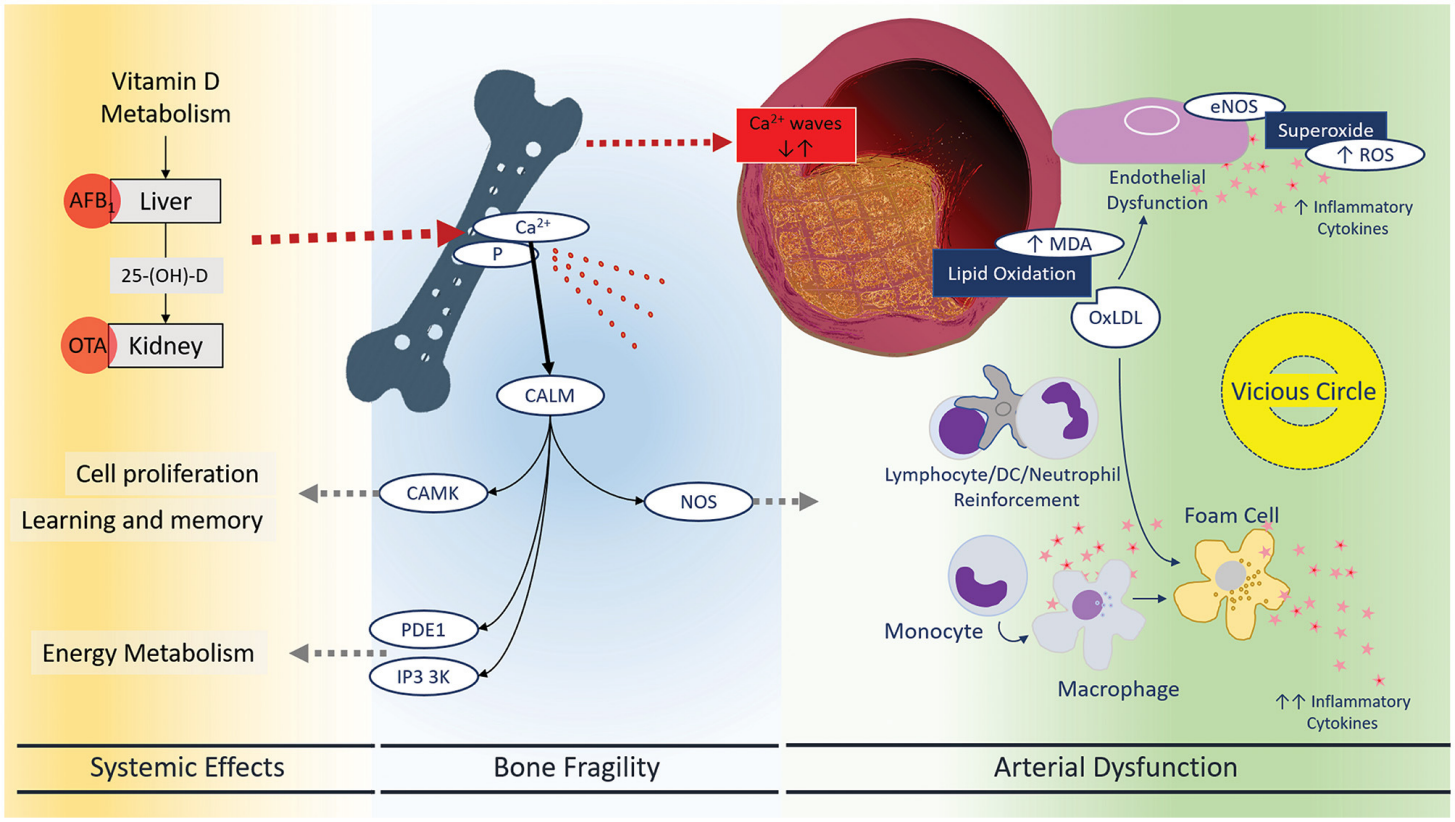
Systemic effects of mycotoxins leading to bone fragility and arterial dysfunction. There has been a meaningful connection between oxidative status and atherosclerotic vascular diseases. Now, mycotoxins are assumed to be an important environmental factor disrupting and or modulating oxidative and immune mechanisms in the blood vessels. Endothelial dysfunction, lipid peroxidation, and foam cell formation are three main characteristics of vascular inflammation: inflammatory responses and immune cell reinforcement. Mycotoxins can restore this vicious circle that may explain the high risk of stroke, heart attack, and PAD. CALM, Calmodulin 1–6, CAMK, calcium/calmodulin-dependent protein kinases, PDE1, phosphodiesterases, IP3 3K, inositol-trisphosphate 3-kinases, NOS, nitric oxide synthase.

The oxidative imbalance may be inevitably enhanced during the atherosclerotic plaque progression as long as the modified lipoproteins and reinforcements possess proinflammatory properties (Figure 1). Of note, environmental triggers are a part of the story when the imbalance between cellular oxidative and antioxidant status becomes a critical underlying factor. Many genes, biomolecules, and immune cells associated with vascular dysfunction are considerably oxidative status-sensitive, the stress enhanced by relatively low mycotoxin levels (Table 2); thereby, a mixture of mycotoxins (Table 3) is an emerging issue in modern toxicological science contributing to different organs' pathobiology (Figure 2).

**Table 2:** The summarized list of stress-related immune pathways modulated by mycotoxins at very low doses; with possibly toxic action at the vascular level.

**Mycotoxin**	**Target cell**	**Altered key process**	**Referenes**
AFB_1_	Neutrophil Monocyte Lymphocyte	Caspase cascade pathway	(55)
	Monocyte	TLR pathway	(56)
	PBMCs		(57)
	DCs		(58)
	Macrophage	Autophagic process Phagocytosis-independent innate immune mechanism (extracellular traps)	(59)
OTA	Epithelial cell	MAPK/ JNK pathway	(60)
T-2	Epithelial cell	Caspase cascade/independent pathways	(61)
	Stem cell	Caspase cascade pathway	(62)
	Neuroblastoma cell	MAPK pathway	(63)
	Monocyte	Differentiation process	(64)
	Macrophage	TLR pathway	(65)
DON	Fibroblast/ Epithelial cell	Caspase cascade pathway	(66, 67)
	Epithelial cell	NF-κB pathway	(68)
	Macrophage	NF-κB/ TLR pathway	(69)
ZON	Macrophage	TLR pathway	(70)
	Epithelial cell	MAPK pathway	(71)
	Leukemic cell	Caspase cascade pathway	(72)

*AFB_1_, aflatoxin B_1_; PBMCs, peripheral blood mononuclear cells; TLR, toll-like receptor; DCs, dendritic cells; OTA, ochratoxin A; MAPK, mitogen-activated protein kinase; JNK, c-Jun N-terminal kinase; DON, deoxynivalenol; NF-κB, Nuclear Factor-Kappa B; ZON, zearalenone*.

**Table 3:** Examples of combined mycotoxins effects disturbing metabolic and immune reactions (focusing on low concentrations).

**Mycotoxin**	**Effective dose, target**	**Effect**	**Major conclusion**	**References**
AFB_1_/ AFM_1_	5–30 μM, murine macrophage	↓ NO production by LPS-stimulated macrophages	Alteration of immune response and ↓defense ability against tumorgenesis	(73)
AFB_1_/ AFB_2_/ AFG_1_/ AFG_2_	1.0, 0.5, 0.25, and 0.25 ng/mL (respectively), bovine PBMCs	↑ TLR-4 mRNA expression level	Signal transduction by TLRs	(74)
AFB_1_/ AFB_2_/ AFG_1_/ AFG_2_	2.0, 1.0, 0.5, and 0.5 ng/mL (respectively), porcine monocyte-derived DCs	↑ Expression of co-stimulatory molecules; CD25 and CD80/86 ↑ Antigen-presenting capacity of DCs	Breakdown of immunological tolerance	(75)
AFB_1_/ AFB_2_/ AFB_2a_/ AFG_1_/ AFG_2_/ AFM_1_	0.1, 1, and 10 pg/mL, rat macrophage	↓ Phagocytosis activity ↓ Intracellular killing and spontaneous superoxide production (The biggest effect by AFB_1_ and AFM_1_ The smallest effect by AFG_1_)	Depressive effect on macrophages and a susceptibility of host to infectious diseases	(76)
AFB_1_/ OTA/ FB_1_/ T-2	1, 3, 10, and 30 μM, human Caco-2 cell	↓ mRNA expression of the tight junction proteins (claudin-3 and occludin)	↑ Paracellular (epithelium) permeability	(77)
AFB_1_/ OTA/ FB_1_	OTA and AFB_1_ (5 and 40 ng/mL) FB_1_ (5 and 40 μg/mL), human mononuclear blood cells	↑ DNA damage and fragmentation ↑ Cell apoptosis ↓ Cell viability	Increased immuno-suppression by chronic exposure, especially in patients with chronic diseases, like cancers, tuberculosis, malaria, and HIV	(78)
OTA/ FB_1_	OTA (5 ng/kg b.w.) FB_1_ (200 ng/kg b.w.), rat kidney and liver	↑ MDA and Protein carbonyls ↓ CAT activity	High potential to induce oxidative damage	(79)
	0.05, 0.5 and 5 μg/mL, porcine kidney epithelial cells	↑ Lipid peroxidation ↓ GSH level ↓ Cell viability	Long-term exposure is an important inducer of immunosuppression and development of chronic renal diseases	(80)
FB_1_/ DON	FB_1_ (110 μg/kg b.w./day) DON (45 μg/kg b.w./day), mice	↑ Triglyceride and total cholesterol ↑ Blood total proteins and creatinine ↑ m5dC level from 3.6 to 7.2% (DNA methylation) ↑ DNA fragmentation	Lipid and lipoprotein (increased serum proteins) metabolism disorders Blood lymphocytes cell deaths	(81)
	3+6 mg/kg, piglet	↓ The number of goblet cells ↑ The number of lymphocytes, plasma cells, and eosinophils ↓ Lymphocytic infiltration ↓ Epithelial cell proliferation ↑ TNF-α, IL-1β, IFN-γ, IL-2, IL-6, IL-12p40, MIP-1β, and IL-10 expression in ileum or jejunum (alone) ↓ Adherent junction protein E-cadherin and occludin in the intestine	Alteration in the intestine and induction of more susceptibility to infections by enteric pathogens	(82)
DON/ ZON	40+12 μg/kg, porcine peripheral blood lymphocytes	↓ CD4^+^ and CD8^+^ lymphocytes	Transiently depletion of immunoregulatory mechanisms (strong effects of mixed AFs)	(83)

*AFB_1_, aflatoxin B_1_; NO, nitric oxide; LPS, lipopolysaccharides; PBMCs, peripheral blood mononuclear cells; TLR, toll-like receptor; DCs, dendritic cells; OTA, ochratoxin A; FB_1_, fumonisin B_1_; HIV, human immunodeficiency virus; MDA, malondialdehyde; CAT, catalase; GSH, glutathione; DON, deoxynivalenol; m5dC, 5-methyldeoxycytidine; TNF-α, tumor necrosis factor-alpha; IL-1β, interleukin 1β; IFN-γ, interferon-gamma; MIP-1β, macrophage inflammatory protein 1-beta; ZON, zearalenone; CD4, cluster of differentiation 4*.

Altering redox balance toward oxidation is a pivotal effect of prolonged exposure to mycotoxins. Circulation is the way by which oxidative agents reach different targets, from endothelial cells to bone marrow/structural cells. Therefore, prolonged exposure to low mycotoxins concentrations activates metabolic feedback between different tissues, affecting, finally, the whole metabolic homeostasis in a fragile condition. Reducing the general antioxidant capacity plus local inflammatory responses can result from exposure to low doses of different mycotoxins.

## Aflatoxin B1

The earliest evidence show ROS system impairment as the underlying mechanism of the AFB_1_ toxic effects at the nanomolar levels (10–1000 nM) (84, 85). The toxic effects of nanomolar AFB_1_ metabolites on oxidative status and immune cells (neutrophils, monocytes, lymphocytes, and dendritic cells [DCs]) in mammals are represented by increased free radicals, inflammatory molecules, and mitochondrial pathways of cell apoptosis (via caspase-3/7/9 activation and ATP depletion) (9, 55, 86–88). The induction of apoptosis caused by 20 ng AFB_1_/mL is due to free radical production (Table 2), the most common sign of intercellular oxidative stress (42, 89). This, in turn, could be considered a potential risk for susceptibility to many chronic diseases. Nonetheless, the knowledge of low-level AFs' oxidative effects increasing the risk of infectious and non-infectious diseases is limited.

The cytochrome P450 (CYP) isoforms, which have key roles in metabolic pathways of AFB_1_, are stimulated via environmentally relevant levels of AFB_1_ (10 and 100 ng/mL) in circulating lymphocytes and monocytes (56). AFB_1_-epoxide strongly binds to the cell macromolecules, triggering more oxidative conditions (90). There is a positive correlation between the induction of CYPs and TLR-4, an essential inflammatory mediator. Highly induced CYPs (CYP1A1 and CYP1B1) by low doses of AFB_1_ in monocytes may lead to a 10-fold upregulation of TLR-4 (Table 2) (56). Thus, ROS production, caused by AFs, drives immune cells to prooxidant/inflammation status, such as autophagy and extracellular trap formation in macrophages, through TLRs (59). The sequential status of oxidative stress is concluded by several folds overexpression of myeloid differentiation primary response 88 (MyD88), TLR-2/4, and CD14 genes in nanomolar concentration of mixed AFs-exposed leukocytes (Table 2) (57), exacerbating immune and metabolic alterations.

Low AFB_1_ levels (50 μg/kg DM) can also induce substantial metabolic alterations involving cell membrane-associated metabolism, the tricarboxylic acid cycle, lipids, glycolysis, amino acid metabolism, serum lipoproteins, and N-acetyl glycoproteins. These are attributable to Cori and Krebs cycle disruptions, energy metabolism and mitochondrial function deregulations, decreased β-hydroxybutyrate and acetate, and increased acetoacetate and acetone production, suggesting the point of low-level AFB_1_-induced β-oxidation of fatty acids and suppressed tricarboxylic acid cycle (91). Together, AFB_1_ is a potent biosynthetic inhibitor affecting the metabolic processes toward the prooxidant status.

We recently showed that AFB_1_ and AFM_1_ at picomolar concentrations could further affect the metabolic pathways by inhibiting insulin production in pancreatic islets *in vitro* (92). In this work, a new framework for the toxicity of mycotoxins has opened. The Aflatoxins, and their metabolites, modulate the endocrine system, either directly or indirectly, via alteration in redox homeostasis. Further studies will scope low and biologically relevant levels of AFB_1_-induced oxidative imbalance translating to infection, metabolic alterations, and chronic inflammation.

## Other Mycotoxins

### Ochratoxin A

Ochratoxin A (OTA) is a well-known potent inducer of ROS and lipid peroxidation in the mammalian system (93). OTA stably accumulates in many tissues and detection of nanomolar OTA (from 0.15 to 9.15 ng/mL) in plasma/serum is not surprising; hence it may be a risk factor for humans, especially following long-term exposure (94). In this context, the oxidative nature of low OTA levels in immune and metabolic processes should be more noticed.

A specific effect of low-level OTA, which is disturbing normal cell homeostasis (Figure 1), is based on OTA's interaction with secondary messengers and carriers, such as mitogen-activated protein kinases (MAPKs) (95). The oxidative stress amplifies the consequences of direct damage to DNA structure and interferes with the DNA repair system in cytotoxic T lymphocyte activity at a low concentration of OTA (96). OTA leads to a time- and dose-dependent induction of apoptosis through disruption in mitochondrial function and B-cell lymphoma extra-large (Bcl-xL) expression in leukocytes and Kit 225 cells. Bcl-xL, a transmembrane molecule in the mitochondria, would inhibit apoptosis by preventing mitochondria oxidative contents, such as cytochrome c efflux to the cytosol and activating caspases. At a low dose (0.5 μM), OTA exposes the cells to mitochondrial transmembrane potential loss and apoptosis (97). Since this toxin has a long half-life, chronic exposure to low-concentration OTA (98) may cause immunosuppression and severe vascular effects.

Surprisingly, in a sub-chronic intoxication model (547.2, 752.5, and 930.3 ng OTA/g kidney tissue in rats within 10, 30, and 60 days), OTA concentrations were higher in serum than in kidneys. Thereafter, oxidative stress and apoptosis are evident through increased MDA formation, lipid peroxidation, and decreased SOD. Increased free radicals level and subsequent mitochondrial membrane damage may accelerate the apoptosis process within 60 days of exposure to OTA (99). OTA-induced loss of Matrix metalloproteinases (MMPs) and antioxidant enzymes demonstrates activation of c-Jun N-terminal kinase (JNK)-mediated caspase3-dependent apoptosis and potentiating inflammatory mediators, such as TNFα (60). The 30-day exposure to low concentrations of OTA (0.05 mg/kg) significantly decreases nuclear factor erythroid 2-related factor 2 (Nrf2) expression, further confirming OTA-mediated prooxidant micro/macroenvironment, exacerbating inflammation (100).

A significant correlation between circulating inflammatory markers, such as C-reactive protein (CRP), and OTA level may be derived from systemic inflammation or mild tissue damage in chronic terms. The hypothesis of OTA-dependent inflammation comes from lipid peroxidation, ROS production, and apoptosis. Concerning the long-term exposure to OTA allied with oxidative subcellular structures (DNA, protein, and lipid), chronic inflammatory conditions, such as autoimmunity, rheumatoid sclerosis, musculoskeletal disorders, and cancer, are a new perspective to warrant further investigations.

### Fumonisin B1 (FB_1_)

There is little evidence about the induction of oxidative and inflammatory processes by prolonged exposure to low levels of FB_1_. The progress in FB_1_ research on oxidative imbalance as the plausible mechanism for pleiotropic toxicities has not been addressed at nanomolar concentrations. The proposed mechanism of action is the inhibition of ceramide synthase by occupying sphingosine and fatty acyl-CoA interactions. Following the inhibition of ceramide biosynthesis, several mechanisms would be correlated with oxidative stress and apoptosis due to immunolocalization of the lower doses of FB_1_ in specific targets, such as mitochondria and nucleus (Figure 1) (101). The lowest FB_1_ concentration (0.5 μM) may raise the rate of cytosolic ROS production and inhibit mitochondrial respiration (102). At the low levels of FB_1_, apoptosis is likely the mechanism of cell death initiating uncontrolled pathways.

The oxidative nature of FB_1_ mediates acute lipid metabolism. The lowest FB_1_ doses (10 and 50 mg/kg) in rats, for a minimum period, changes the main cellular polar lipid fractions and the fatty acid (FA) composition of the hepatic mitochondrial phospholipids. A decreased phosphatidylcholine and phosphatidylethanolamine decreased the polyunsaturated/saturated fatty acids (FA) ratio which suggests that FB_1_ impairs the membrane fluidity (103). Also, the proportion of fatty acids decreased in the phospholipid fraction, modifying the hepatocellular membrane lipids, indicates the acute membrane-damaging effect of FB_1_. C_22_ N_3_ polyunsaturated FA (PUFA) shows a proportional decline because of very high sensitivity to oxidative stress and lipid peroxidation reflected by MDA and glutathione (GSH). The hepatic GSH level, a well-known marker of antioxidative capacity, decreases under FB_1_ exposure. And one might hypothesize that FB_1_ targets and modifies the membrane phospholipids to generate hydrogen peroxides. Therefore, lipid peroxidation in the FB_1_-induced stress is probably reflected by increased lipid-associated parameters, including hepatic lipoprotein secretion, total cholesterol, and MDA and GSH levels in blood plasma (103).

**Figure 3:**
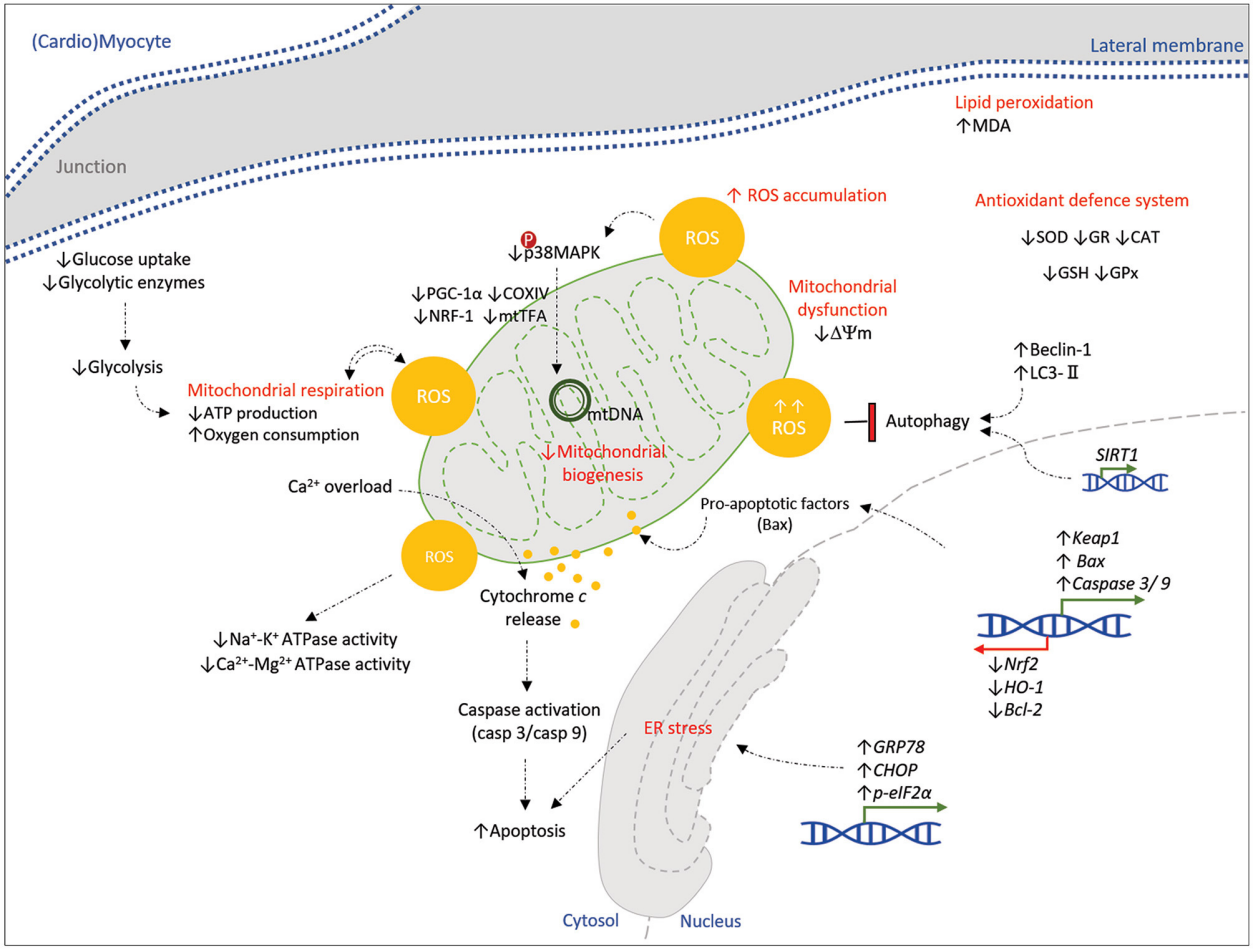
Schematic representation of the molecular pathways affected by mycotoxins in a (cardio)myocyte. Mycotoxins (AFB_1_, OTA, T-2, DON, and ZON) show toxicity on a (cardio)myocyte, principally by excessive oxidative stress and ROS accumulation. Alterations in mitochondrial bioenergetics, mitochondrial dysfunction, transcription of stress/apoptotic genes, and ER stress orchestrate the cell damage and apoptosis. Dysregulation of the antioxidative system and protective pathways (autophagy) would enhance these mechanisms of toxic myopathy. Down- and upstream signaling pathways that might be affected by mycotoxins in a myocyte are still unknown to be represented here; hence it is highly suggested to be determined by further studies. Green and red arrows (blocked line) indicate activation and inhibition, respectively.

Exposure to even permissible levels of FB_1_ would induce lipid peroxidation and redox imbalance due to mitochondria involvement, which could lead to sequential proinflammatory micro/macroenvironment in various cells and organs (104). The effects, naturally, will be more pronounced in muscles and neurons due to their significant dependence on mitochondria (Figure 3). It is needed to do more on the lower or nanomolar levels of FB_1_ to elucidate the molecular mechanism.

### Trichothecenes (T-2)

T-2 toxin is lipophilic and easily absorbed through the skin, gut, and pulmonary mucosa. Exposure to a sub-lethal dose of T-2 toxin refers to several hematologic and immunotoxic effects targeting immune cells, macrophages, and peripheral blood B and T lymphocytes (105). Hematopoietic and lymphoid tissues are susceptible to T-2 toxin (106). It causes apoptotic death in highly proliferating cells, such as lymphocytes (107) and intestinal crypt epithelial cells (108). Nanomolar T-2 and its major metabolite (HT-2) may account for redox imbalance in the circulation (Figure 1) and intracellular spaces underlying apoptosis induction (109).

Therefore, oxidative stress is the suggested mechanism of T-2 toxicity as LC_50_ of 10 ng/mL induces apoptosis in HeLa cells through caspases dependent and independent pathways (61). Even at a concentration <2 ng/mL, DNA fragmentation and apoptosis occur through the involvement of mitochondrial apoptogenic factors, Bcl-2-associated X (Bax), B-cell lymphoma 2 (Bcl-2), cytochrome *c*, apoptosis-inducing factor (AIF), and caspases 9, 3, and 7 (Table 2) (42, 62). Then, increased ROS and MDA levels, associated with loss of intracellular GSH, SOD, and catalase (CAT) activities, prove the evidence of oxidative stress as the main mechanisms of 1 and 2 ng T-2 toxin/mL-induced apoptosis (110).

T-2 toxin activates apoptotic processes through the ROS-mediated MAPK signaling pathway (Table 2) (63). Exposure to high nanomolar levels (40 nM) causes a significant time-dependent increase in ROS and MMP loss in GH3 cells (111). The implication of T-2 toxicity at nanomolar concentrations can be significantly reduced by protecting the mitochondrial activity (112).

Monocytes and macrophages are privileged targets of T-2, enough to control the principal mediators of inflammation (64). The potential mechanism of this disturbance in monocyte differentiation has not been explored.

A pre-exposure to nanomolar T-2 concentrations reduces nitric oxide (NO) production and cell viability in the porcine alveolar macrophages activated by TLR-4 and TLR-2/6 agonists (Table 2) (65). Then, TLRs signal inflammatory pathways and metabolic inflammation. It can explain how metabolic imbalance underlying low-level inflammatory processes resulting from T-2 would be the common characteristic of chronic conditions such as vascular dysfunction and sarcopenia.

### Deoxynivalenol (DON)

DON or vomitoxin disturbs oxidative status in rainbow trout at concentrations below the limit fixed by the European Commission (113). In animals fed with low-dose DON, prooxidant and antioxidant parameters analyses indicate changes in SOD, GPx, and CAT activities in erythrocytes and the blood (114).

The metabolomics data can confirm the role of oxidative stress and metabolic alterations in cellular processes, including redox homeostasis, energy balance, membrane integrity, and lipid metabolism by DON (Figure 1) (115). Peroxidation of phospholipids and perturbation of membrane integrity are expected in exposed cells. It triggers lipid peroxidation (high MDA levels), responsible for membrane and DNA damage in human lymphocytes (116). Nanomolar of DON causes antioxidants (GSH) depletion and oxidative DNA damage, leading to reduced expression of heme oxygenase-1 (HO-1) (a homeostatic and protective agent against cellular stress) and loss of DNA reparative potential. Thus, it potentially induces genotoxicity and apoptosis in human peripheral blood lymphocytes (42, 116).

The intracellular apoptotic signals of DON are also explored on epithelial cells. Activation of the caspase cascade system, such as caspase-3, caspase-8, caspase-9, and poly (ADP-ribose) polymerase, and the increased level of P53, ROS, and the ratio of Bax/Bcl-2 contribute to loss of MMP in epithelial cells (66). Following 250 ng/mL DON exposure, the intracellular ROS and ribotoxic stress drive the expression of inflammatory genes, NF-κB and cyclooxygenase-2 (COX-2) (Table 2) (68). In macrophages, this DON-mediated effect on NF-κB is stimulated by MyD88-dependent TLR signals. Hence, DON poses a risk of an oxidative imbalance in the organelles, such as ribosome and endoplasmic reticulum (ER), leading to T-cell activation and apoptosis (117). Characterization of the modes of action of DON, therefore, formulates the potential mechanisms of DON toxicity on immune and endothelial cells in the blood.

### Zearalenone (ZON)

ZON is known for its robust estrogenic activity because it competes with 17β-estradiol to bind to cytosolic estrogen receptors (118). But, oxidative damage has been recently documented as an initiating event responsible for different genotoxic and cytotoxic effects of ZON (Figure 1) (119). Non-cytotoxic concentrations impairs cell viability through oxidative DNA damage, GSH depletion, and stress-responsive genes (Hsp70 and Hsp90) induction (119). Mitochondrial oxidative stress, plasma membrane permeabilization, and apoptosis are induced by 3 and 100 nanomolar (120).

Low levels of ZON lead to an imbalance in immune cells and pro-/anti-inflammatory cytokines. It changes blood lymphocyte populations and proliferative capacity, with a significant increase in IFN-ɤ levels (83). Co-exposure to ZON metabolites might cause more substantial adverse effects on the viability and expression of inflammatory factors, TLR ligand stimulation, IL-1β, TNFα, IL-6, and IL-8 (121). MAPK pathways involving extracellular signal-regulated kinases (ERKs), p38, and JNK signaling are essential in transducing extracellular stimuli; that is, oxidative signals derived from ZON (71). However, the exact molecular mechanisms of ZON toxicity inducing inflammatory reactions have not been investigated. The cytotoxic effects of ZON are, in part, thought to be correlated with its apoptotic potential by activation of mitochondrial cytochrome *c*, ROS production, induction of caspase-3/8, and ER stress in human leukemia cells (Table 2) (72). Regarding changes in blood metabolic profile associated with long-term exposure to low doses of ZON, it would represent a probable reason behind vascular damage.

## Vascular Specific Disruptions

Among mycotoxins, AFs are more studied due to their higher potential to disrupt. Mycotoxins share many mechanisms of action; therefore, here, the discussion will follow with AFs. The acute effect of AFs on the cardiovascular system is vascular fragility and thus hemorrhage and teratogenicity. Metabolic alterations and inflammatory conditions might occur due to AFs exposure to oxidative stress leading to lipid peroxidation and oxidative DNA damage (Figure 1) (122). Lipid peroxidation is the most destructive process in cells associated with excessive ROS production. The high or moderate increase in ROS seems detrimental for myocardial cells, while its physiological level is necessary for normal cell signaling (123).

Intraperitoneal administration of AFB_1_ as a single dose of 2 mg or 17 μg/kg increases markers of oxidative damage in the heart, such as thiobarbituric reactive substances, like MDA, and calcium (Ca)^2+^ levels (124). Cardiomyocytes, and more generally all muscle cells, are specifically susceptible to oxidative modifications and free radicals because the antioxidant defense mechanisms are very limited in these cells compared to other types of cells in the body (125). Indeed, the susceptibility of cardiomyocytes and vascular muscles to oxidative damage under aflatoxicosis is due to the significant reduction in total antioxidant capacity, ATPase, and antioxidant enzymatic and non-enzymatic activities, including SOD, CAT, glutathione reductase (GR), GPx, GSH, and vitamins C and E (123).

In addition, AFB_1_ interferes with cellular energy supply in myocardial cells (126). Glycolysis is also one of the most important metabolic processes participating directly in ATP production to meet cellular energy demands in the heart. Myocytes need continuous ATP production as it cannot be restored (127, 128). AFB_1_ alters such bioenergetic sources, increasing myocytes' susceptibility to metabolic stress and affecting myocardial contractile performance (126). A single dose of 1 mg AFB_1_/kg can inhibit glycogen synthesis, glucose uptake (a decrease in glucose contents of myocardial cells), and cardiac glycolysis in rats. Then, glycolytic enzymes, hexokinase, glyceraldehyde 3-phosphate dehydrogenase (GAPDH), phosphoglucose isomerase (PGI), and lactate dehydrogenase (LDH), as well as the rate of glycolysis, are declined by AF exposure (126).

As energy producers (90% of ATP), mitochondria have attracted much interest regarding impairment of mitochondrial activity which may result in cardiomyopathy. In rat cardiomyocytes, low doses of AFB_1_ result in mitochondrial damage through disruption in mitochondrial membrane and cristae (129). Mitochondrial dysfunction and expression of apoptotic proteins, such as active caspase-3, Bax, and Bcl-2 caused by low levels (0.75 mg/kg) of AFB_1_ can finally cause severe cardiomyocyte dysfunction (129). It could be a hidden danger because cardiomyocytes' renewal is extremely low in humans (130). However, the precise mechanisms of AFB_1_ and cardiomyocytes' apoptotic pathways are not precise.

High NO concentration and pro-inflammatory cytokines, TNFα and IL-1α by AFB_1_ ingestion (131), can also inhibit mitochondrial ATP production and stimulate cardiomyocytes apoptosis (132). In the low range of concentrations, 0.5, 1.0, 2.5, and 5.0 μmol/L of AFB_1_, mitochondrial functions are determined by glutamate/malate and succinate-driven respiration. In a concentration-dependent manner, glutamate/malate-driven respiration is significantly uncoupled, increasing the state of respiration significantly vs. a decrease in broiler cardiomyocytes (BCMs) (133). Hence, AFB_1_ may uncouple the mitochondrial respiration and oxidative phosphorylation in BCMs. AFB_1_ results in a significant and concentration-dependent increase in intracellular ROS production in BCMs because a main source of ROS can be mitochondrial respiratory chain uncoupling (133). High levels of mitochondrial ROS and membrane lipids peroxidation exhibit mitochondrial oxidative stress-induced apoptosis in BCMs. On the other hand, this toxicity response to AFB_1_ is proven by a significant increase in LDH and cardiac troponins leakage from BCMs, as sensitive markers for acute cardiac damage (133). It is well-known that the antioxidant defense system, including antioxidant enzymes and sensitive transcription factors, tries to preserve the redox balance (134). Considering this condition, there is a significant decrease and increase in mRNA expression of SOD and Nrf2 genes, respectively, in BCMs by exposure to AFB_1_ (133). So, the myocardium of rats indicates patchy necrosis with a surrounding inflammatory reaction within the first 4 days of AFB_1_ exposure.

The histopathological examination of ventricular muscle sections in rabbits orally administered at a dose of 30 μg AFB1/kg revealed nuclear pyknosis and peripheralization, sarcoplasmic vacuolation, and myofibrils degeneration with the use of minimization of disrupted intercalated disks and congestion and dilatation blood vessels, in addition to mononuclear cellular infiltration. Edema distribution among myocardial fibers illustrates severe cardiotoxicity and subsequent heart damage by AFs. Figure 3 summarizes mycotoxins toxicity in a (cardio)myocyte at the molecular level. According to similar data in Table 4 for cardiotoxicity induced by other common mycotoxins at low and high levels, the chronic oxidative stress and damage would be concluded by long-term exposure to different levels of mycotoxins. Although this condition represents cardiac cell damage and heart failure *in vitro* and *in vivo*, there is still a need to do large-scale investigations.

**Table 4:** The cardiotoxicity of mycotoxins.

**Mycotoxin**	**Effective dose, target**	**Effect**	**Major conclusion**	**Ref**.
OTA	289 μg/kg, myocardial tissue of rat	Histopathological changes on myocardial tissue; Extensive cytoplasmic vacuole formation Necrosis of the myocytes Dissolution of the nucleus Clumped fibers Fibrinolysis Swollen myocardial fibers Small hemorrhagic areas and hyperaemic vessels	Heart damage (reduced by the antioxidant effect of melatonin)	(135)
	0.1 mL of OTA 5 mg/kg b.w., mice	↓ Heart weight and rate ↓ SOD, CAT, and GSH ↑ Cardiac enzymes (CK, CK-MB, and LDH) ↑ MDA level ↑ Keap1, Bax, caspase 3 and 9 expressions level ↓ Nrf2, HO-1, and Bcl-2 expression level	Myocardial injury; mitochondria-mediated apoptosis pathway; (protection by Keap1-Nrf2 signaling pathway)	(136)
T-2	0.5 ng/mL, murine embryonic stem cells (embryoid bodies)	↑ ROS accumulation ↑↓ Phosphorylation of p38 (time-dependent) ↓ Mitochondrial number ↓ Mitochondrial biogenesis-related proteins (PGC-1α, NRF-1, mtTFA, and COXIV)	Inhibition of cardiac differentiation; via p38MAPK- and ROS-mediated mitochondrial pathway	(137)
	0.23 mg/kg, cardiac tissue of rat	Cardiac histopathology; Myofibril degeneration Hemorrhages Glycogen distribution Accumulation of neutrophils, macrophages and mast cells Mast cells degranulation	Progressive myocardial injuries (cardiomyocytes' lysis and loss of cross-striation) by long exposure (28–60^th^ day)	(138)
	0.125 and 0.25 (-1) μM, primary cardiomyocytes of rat	Autophagy induction; ↑ LC3-II and Beclin-1 levels ↓ Cardiomyocytes viability ↑ LDH release ↑ Cleaved caspase 3 ↑ ROS generation ↓ GSH level and SOD activity (significant with lower dose: 0.125) ↑ MDA level (significant with lower dose: 0.125) ↓ Na^+^-K^+^-ATPase activity and Ca^2+^-Mg^2+^-ATPase activity ↑ GRP78, CHOP, and p-eIF2α mRNA levels (ER-stress)	Antioxidant (selenium) deficiency decreases autophagy activity protecting cardiomyocytes and aggregates cardiomyocyte injury through ER stress	(139, 140)
T-2/ DON	6.0×10^−6^ and 6.0×10^−5^ μM (T-2) 0.39 and 0.78 μM 1.56 and 3.13 μM (DON), primary cardiomyocytes of rat	Inhibition of ATP-linked OCR Inhibition of bioenergetics reserve capacity	Inhibition of mitochondrial ETS function associated with oxidative stress in cardiomyocytes	(141)
ZON, α- and β-ZOL	20-100 μM, H9c2 cell line (embryonic rat heart)	Autophagy induction; ↑ LC3-II and Beclin-1 levels (before the onset of apoptosis) Activation of the mitochondrial apoptotic pathway ↑ ROS generation Loss of mitochondrial transmembrane potential (ΔΨm) Caspases activation Increased cell death	Higher level of ROS and oxidative stress by long-term exposure (24 h) to ZON and its derivatives overcomes a cardioprotective mechanism (SIRT1-mediated autophagy)	(142)

*OTA, ochratoxin A; SOD, superoxide dismutase; CAT, catalase; GSH, glutathione; CK, creatine kinase; CK-MB, creatine kinase isoenzyme; LDH, lactate dehydrogenase; MDA, malondialdehyde; Bax, Bcl-2-associated X; Nrf2, nuclear factor erythroid 2-related factor 2; HO-1, heme oxygenase 1; Bcl-2, Bcl-2, B-cell lymphoma 2; ROS, reactive oxygen species; PGC-1α, peroxisome proliferator-activated receptor coactivator-1 alpha; NRF-1, nuclear respiratory factor 1; mtTFA, mitochondrial transcription factor A; COXIV, mitochondrial respiratory chain complex IV; p38MAPK, p38 mitogen-activated protein kinase; LC3-II, microtubule-associated protein light chain 3 II; ATPase, adenosine triphosphatase; GRP78, glucose Regulated Protein 78 kDa; CHOP, CCAAT enhancer-binding protein homologous protein; p-eIF2α, phosphorylated α subunit of eukaryotic initiation factor 2; ER, endoplasmic reticulum; DON, deoxynivalenol; OCR, oxygen consumption rate; ETS, electron transport system; ZON, zearalenone; ZOL, zearalenol; SIRT1, sirtuin 1*.

## Effects on Systemic Metabolism and the Bone

Mycotoxins affect different cells throughout their travel in circulation. As aforementioned, the effects on redox balance favoring more oxidation can reduce the antioxidant capacity in the circulation and damage protective mechanisms. Bone homeostasis strictly depends on the correct function of metabolic pathways and the endocrine system, particularly vitamin D. There is experimental evidence that low-level AFs supposed to change vitamin D and parathyroid hormone (PTH) metabolism (143).

Also, vitamin D metabolism is consistently associated with the modulation of vascular tone. It is correlated with atherogenic blood lipid profiles, such as total cholesterol, triglycerides, and LDL/HDL-cholesterol, as risk factors for cardiovascular diseases (144). Supplementation studies evident the vitamin D deficiency in the development of atherosclerosis at the site of the blood vessels (145). It is an atherogenic factor influencing adhesion molecules, ECs activation, and VSMC proliferation. Simultaneously, vitamin D deficiency is associated with oxidative stress and inflammation involving immune cells, particularly monocytes and macrophages (144). The 22-oxacalcitriol is a vitamin D analog that can suppress the high expression levels of p22phox and NADPH oxidase enzyme (generating superoxide) and improve eNOS coupling, thereby reducing ROS production oxidative stress in the vasculature (146).

Considering the mechanism of action, vitamin D, in the active form of 1,25-(OH)_2_-D, binds to nuclear vitamin D receptor (VDR), membrane-bound and cytoplasmic receptors. These receptors are considerably expressed in all cells playing in atherosclerosis, such as ECs, VSMCs, and immune cells. Hence, vitamin D might regulate a wide range of physiological and pathological processes, including vascular cell growth, migration, and differentiation, immune response modulation, and inflammatory pathways in the blood vessels (147).

Naturally occurring mycotoxins might indirectly affect bone and skeletal apparatus hemostasis via interference with vitamin D metabolism. Increasing low-level AF intake significantly decreases bone mineralization parameters, such as tibia breaking strength (TBS) and the percentages of Ca and P in the tibia, associated with increased PTH and decreased PTH 1,25-dihydroxycholecalciferol. These adverse effects on broilers' P metabolism and bone mineralization might relate to the vitamin D metabolism (143).

It has been reviewed elsewhere that certain mycotoxins, singly and severally, including AFB_1_, OTA, T-2 toxin, and FB_1_, can affect bone growth strength and cause bone fragility (148). As a crucial hormone in Ca bone's homeostasis and metabolism, vitamin D has been essential for bone health (149). Mycotoxins cause a metabolic situation, such as reducing circulating Ca and P content in the blood, eventually reducing bone strength (148).

Notably, the expression of VDRs was significantly downregulated by the natural occurrence of AFB_1_ (at nM concentrations) in osteosarcoma cell line SaOs-2. It could suggest that naturally occurring mycotoxins that are toxic toward VDRs can potentially interfere with vitamin D's action on Ca-binding gene expression in tissues (150). Therefore, the unexpected toxicity of the AFB_1_ toward the VDRs proposes indirect effects of combined mycotoxins on the cardiovascular system.

T-2 toxin targets bone and exposure to T2 may associate with several bone diseases. Indeed, the C57BL/6 T-2 toxin suppresses Wnt/β-catenin signaling and expression of downstream target genes and increases autophagy and apoptosis (151).

Bone marrow can be a sensible target for mycotoxins action. For instance, mesenchymal stem cells (MSCs) are susceptible to oxidative stress that can induce DNA breaks and genomic instability (152). Exposure of pregnant rats to FBs impairs bone metabolism of the offspring via alteration of the osteoprotegerin (OPG)-to-RANκL (receptor activator of nuclear factor κB ligand), possibly driven by immunological alterations (153), and by affecting the expression of remodeling enzymes [MMPs and their tissue inhibitors (TIMPs)] and angiogenic factors [vascular endothelial growth factor (VEGF)] (154).

Besides this evidence, calcium-phosphorous metabolism, vitamin D action, and bone turnover emerge as important targets of mycotoxins' effects. Therefore, it may be of interest to enhance the knowledge of these aspects.

## Conclusion and Future Directions

Acknowledging potential mechanisms responsible for releasing the peroxidative decomposition products, for example, MDA, in the arteria would open new perceptions of biochemical and cellular events in arterial dysfunction and strategies for intervention. Oxidative stress represents a long-term reaction against some stimuli, which is characterized by a long-lasting inflammatory condition. Therefore, it becomes a unifier of various chronic diseases, such as artery diseases, considering the key facts. Activating mitochondrial permeability transition leads to potential loss and the release of cytochrome *c* upregulates the caspase-3 and−9 (62). Excessive ROS production under the pathological condition cannot be efficiently inhibited by the protective antioxidant mechanisms, leading to a state of lipid and DNA peroxidation.

Mycotoxins-assisted oxidative imbalance, mitochondrial dysfunction, and cytotoxicity are unusual mechanisms of action in biological systems. Due to the widespread presence of mycotoxins as contaminants frequently occurring in feed and food, it is likely that the oxidative aspect of mycotoxins individually could also be enhanced by subchronic coexposure to all of them. As represented in Table 3, chronic exposure to combined mycotoxins may have additive or synergistic impacts on the oxidative and immune players. It is necessary to fully understand to what extent the oxidative status in this microenvironment, involving an essential component of the human cardiovascular system, may be responsible for priming immune disruptions, artery diseases, and cardiomyopathies in individuals exposed to the combined mycotoxins. Future studies may also indicate the importance of antioxidant applications in human health to benefit from the promising therapeutic and preventive strategies in people at risk of diseases.

Apart from the oxidative nature of mycotoxins, their intracellular molecular hits remain inconclusive. There is little knowledge regarding the putative proteins that can be targeted directly with these small toxins and/or their metabolites. The lack of knowledge is more remarkable considering the other groups of mycotoxins, such as masked and minor mycotoxins, all of which are unregulated. Further research is needed to address this critical piece of understanding.

Mycotoxins' indirect systemic effects throughout the unfavorable influence on calcium signaling and VitD metabolism (Figure 2) involve key organs responsible for maintaining homeostasis. In conditions with lowering the VitD precursors due to mycotoxins' effects on intestinal cells or hepatocytes, the bone will release the calcium into the circulation. Calcium release and reabsorption are cycles that dynamically keep the general homeostasis; however, dysregulation of this dynamic can create a pulse of high Ca concentration in circulation promoting calculations in arterial walls. Hence, lowering the Ca content of the bone leads to osteoporosis susceptibility and/or bone fragility. The unusual release of Ca from the bone activates downstream Ca/calmodulin-dependent cascades, affecting cell survival, inflammation, and energy metabolism. The discovery of proteins affected by most and/or the possible more susceptible protein isoforms can be the next step in this research field.

## Author Contributions

AM and GL: conceptualization, writing, editing, and final approval. SS: writing, editing, and final approval. JM: conceptualization, editing, and final approval. All authors contributed to the article and approved the submitted version.

## Funding

The works of JM and AM, cited in this review, have been supported by grants from the Ferdowsi University of Mashhad, the University of Tehran, and the Iranian National Elite Foundation. AM has received support from Italian Ministry of Health, Ricerca Corrente Program, to the IRCCS Istituto Ortopedico Galeazzi.

## Conflict of Interest

The authors declare that the research was conducted in the absence of any commercial or financial relationships that could be construed as a potential conflict of interest.

## Publisher's Note

All claims expressed in this article are solely those of the authors and do not necessarily represent those of their affiliated organizations, or those of the publisher, the editors and the reviewers. Any product that may be evaluated in this article, or claim that may be made by its manufacturer, is not guaranteed or endorsed by the publisher.
